# Manipulation of both virus- and cell-specific factors is required for robust transient replication of a hepatitis C virus genotype 3a sub-genomic replicon

**DOI:** 10.1099/jgv.0.000932

**Published:** 2017-10-06

**Authors:** Lorna Kelly, Anjna Badhan, Grace C. Roberts, Jean Lutamyo Mbisa, Mark Harris

**Affiliations:** ^1^​ School of Molecular and Cellular Biology, Faculty of Biological Sciences, University of Leeds, Leeds, LS2 9JT, UK; ^2^​ Astbury Centre for Structural Molecular Biology, Faculty of Biological Sciences, University of Leeds, Leeds, LS2 9JT, UK; ^3^​ Public Health England, 61 Colindale Avenue, London, NW9 5EQ, UK

**Keywords:** hepatitis C virus, genotype 3, sub-genomic replicon, culture adaptive substitution, direct acting antivirals

## Abstract

Hepatitis C virus (HCV) genotype (GT) 3 is the second most prevalent of the seven HCV genotypes and exhibits the greatest resistance to the highly potent, direct-acting antivirals (DAAs) that are currently in use. Previously a stable cell line harbouring the S52 GT3 sub-genomic replicon (SGR) was established, but this SGR was unable to robustly replicate transiently. As transient SGRs are a critical tool in the development of DAAs, and in the study of viral resistance, we sought to establish a transient SGR system based on S52. Next-generation sequencing was used to identify putative culture-adaptive substitutions that had arisen during long-term selection of the S52 SGR. A subset of these substitutions was built back into the S52 SGR in the context of a CpG/UpA-low luciferase reporter, with a single point mutation in NS4A conferring the greatest replication capability upon S52. Modification of the innate immune-sensing pathways of Huh7.5 hepatoma cells by expression of the parainfluenza virus type 5 V protein and SEC14L2 resulted in a further enhancement of S52 replication. Furthermore, this transiently replicating SGR showed genotype-specific differences in sensitivity to two clinically relevant NS5A DAAs. In conclusion, we report that a single substitution in NS4A, coupled with host cell modifications, enabled robust levels of transient replication by the GT3 S52 SGR. This system will have beneficial uses in both basic research into the unique aspects of GT3 biology and drug discovery.

## Abbreviations

DAA, direct acting antiviral; DCV, daclatasvir; Feo, neomycin phosphotransferase/firefly luciferase; GT, genotype; HCV, hepatitis C virus; IFN, interferon; IRES, Internal ribosome entry site; JFH, Japanese Fulminant Hepatitis; LDV, ledipasvir; NGS, next generation sequencing; NS, non-structural; SEC14L2, SEC14 like lipid binding 2; SGR, sub-genomic replicon; SOF, sofosbuvir; SVR, sustained virological response.

## Introduction

Approximately 170 million people worldwide are estimated to be chronically infected with hepatitis C virus (HCV) [1], leading to fibrosis, cirrhosis, liver failure and hepatocellular carcinoma (HCC) [2]. To date seven genotypes have been identified [3], within which a number of subtypes show different global distribution patterns. Genotype (GT) 1 is most prevalent worldwide, with GT1b being the most common genotype in northern Europe. GT3 is the most common GT in low- to middle-income countries (LMIC), accounting for 44 % of cases. In particular, 70 % of HCV infections in South Asia (Pakistan, India and Thailand) are GT3, and it is thought that the global dissemination of GT3 is partially due to population migration from this area of the world. Consistent with this, GT3 is prevalent in parts of Western Europe, especially the UK where it accounts for 44 % of HCV cases. Overall, it has been estimated that over 50 million people are infected with HCV GT3 [1].

Recent years have seen the development of potent, direct-acting antivirals (DAAs) to treat HCV. These compounds target the virus NS3 protease (e.g. simeprevir), NS5B RNA polymerase [e.g. the nucleoside analogue sofosbuvir (SOF)] and NS5A [e.g. daclatasvir (DCV) or ledipasvir (LDV)]. DAAs are better tolerated than the previous interferon-α (IFNα)-based regimens, with sustained virological response (SVR) rates of close to 100 % for GT1 patients without cirrhosis routinely reported [4]. However, IFNα-free therapies are less effective against GT3. GT3 patients who are non-cirrhotic and treatment-naive exhibit SVR of 67–96 % following a 12–16-week SOF/ribavirin regimen, with added benefits of 24 weeks of treatment (86–91 %) and inclusion of DCV (96 %). Patients with compensated cirrhosis who are treatment experienced typically show SVR rates of 21–62 % even in trials including combination therapy of DCV and SOF [5–11].

GT3 infection is associated with a more rapid progression of liver disease and a direct correlation with metabolic syndrome. This leads to a higher incidence of insulin resistance, steatosis (fatty liver) and hepatocellular carcinoma compared to other GTs [12]. This is of increasing concern given the results of clinical trials showing that HCV patients with compensated cirrhosis respond less well to all-oral DAA regimens and that GT3 patients with cirrhosis respond minimally to treatment [13].

Development of new therapies has relied upon the sub-genomic replicon (SGR) system, which was first reported for GT1b [14]. Subsequently the efficiently replicating GT2a isolate JFH-1 [15] has become widely used throughout the field of HCV research. Initially the SGR constructs contained a neomycin phosphotransferase selectable marker allowing the establishment of stable cell lines harbouring the SGR. Critical for the development of DAAs was the availability of a transient, luciferase-based, SGR, but such a system does not yet exist for GT3. Three separate GT3 SGRs derived from two different patient isolates have been reported, but neither of these shows robust levels of replication in short-duration, transient experiments. The S52 SGR replicates to high levels during selection and generates levels of HCV RNA comparable to JFH-1, but has not been demonstrated to replicate transiently using a luciferase reporter [16]. The S310/A SGR has been shown to replicate transiently but luciferase levels were several orders of magnitude lower than the input translation [17], and another S52-based SGR which was culture-adapted in Huh7-Lunet cells showed detectable levels of replication at 7 days post-transfection [18].

The majority of studies with GT3 have thus far used chimeric SGRs, in which fragments of GT3 isolates, or consensus sequences, were used to replace the corresponding coding regions in efficiently replicating GT1 or GT2 backbones. These have been used to show differential sensitivity to NS5A and NS5B inhibitors *in vitro* of GT3 sequences compared to wild-type controls [19–21]. Recombinant SGRs are limited in that they do not allow study of the cognate interactions between viral proteins in the replication complex, and this may provide a hindrance to development of combination therapies. An intact (non-chimeric) GT3 SGR that replicates transiently would be of benefit to understanding the baseline resistance of GT3 to the DAAs, and for development of new DAAs with efficacy against GT3.

To this end we report here the establishment of a robustly replicating transient GT3 SGR. This required both SGR modifications, including additional culture-adaptive mutations, and host cell alterations such as expression of inhibitors of the innate antiviral response.

## Results

### The S52 SGR does not replicate transiently but can establish stable SGR-harbouring cells following selection

**Fig. 1.**
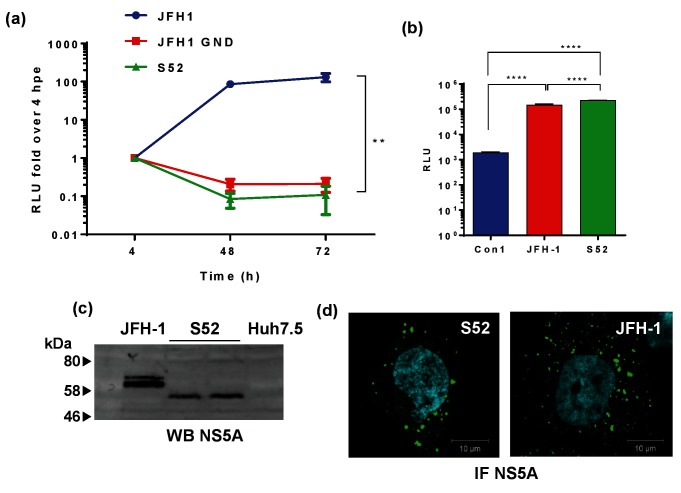
Assay for replication of S52 SGR. (a) Transient replication of the S52 SGR (AII culture adapted variant) [16] compared to either wild-type or GND mutant JFH-1 (GT2a). Two micrograms of the indicated RNA transcripts were electroporated into Huh7.5 cells and harvested for luciferase assay at the indicated time points. Relative luciferase units are expressed as the ratio to 4 hpe. Error bars show standard error of the mean of three experimental repeats. (b) S52 SGR RNA was electroporated into Huh7.5 cells and selected with 0.5 mg ml^−1^ G418 from 48 hpe. Surviving colonies were pooled into a polyclonal population of SGR-harbouring cells. Luciferase activity was measured in 8×10^3^ cells and presented as absolute values compared to Con1- and JFH-1 SGR-harbouring cell lines. (c) Western blot analysis of NS5A expression in JFH-1- or S52 SGR-harbouring cells. (d) S52- and JFH-1 SGR-harbouring cells were immunostained for NS5A (green) using a sheep polyclonal anti-NS5A serum and nuclei using DAPI. **P≤0.01, ****P≤0.0001.

The S52 GT3a SGR [16] was assembled from a consensus full-length DNA clone of the S52 clinical isolate [22], and consists of a bicistronic construct containing a neomycin phosphotransferase/firefly luciferase (Feo) reporter under the translational control of the HCV internal ribosome entry site (IRES), together with the NS3-5B coding region under the control of an EMCV IRES. Importantly it was engineered to contain three culture adaptive substitutions (T1056A, T1429I and S2204I by H77 numbering) and was thus called S52(AII) [16]. For clarity it will be named S52 hereafter. To test for transient replication of the S52 SGR, *in vitro* transcribed RNA was transfected into Huh7.5 cells by electroporation and compared with the GT2a JFH-1 SGR. As shown in Fig. 1, the S52 SGR was indistinguishable from the GND (polymerase-inactive) mutant of JFH-1 and did not replicate to detectable levels in Huh7.5 cells.

As the S52 SGR has been reported to establish stable replicon-harbouring cells [16], we sought to reproduce this observation. *In vitro* transcribed S52 SGR RNA was electroporated into Huh7.5 cells and selected with G418 for three weeks, after which time a small number of colonies of stable SGR-harbouring cells were obtained. These cells were pooled into a polyclonal population and maintained under G418 selection. As can be seen in Fig. 1(b), these cells exhibited steady-state levels of firefly luciferase activity that were comparable to that in cells stably harbouring the corresponding JFH-1 SGR and considerably higher that the Con1 (GT1b) SGR. NS5A expression was analysed by WB, with only a single species of NS5A being observed in the S52 SGR cells consistent with the presence of the S2204I substitution that abrogates hyperphosphorylation (Fig. 1c). Lastly the S52 SGR-harbouring cells exhibited a similar distribution of NS5A to JFH-1 SGR-harbouring cells, with the protein being found in punctate structures located throughout the cytoplasm (Fig. 1d).

### Additional putative culture adaptive substitutions are acquired during selection of stable S52 SGR-harbouring cells

The observation that the S52 SGR did not exhibit detectable transient replication, yet was able to establish stable G418-resistant cells, suggested that it might have acquired additional substitutions that supported higher-level replication. To test this hypothesis, SGR RNA in these cells was amplified by RT-PCR and subjected to next-generation sequencing (NGS). Analysis of the data revealed the presence of nine single nucleotide substitutions at greater than 20 % variant frequency. Seven of these were non-synonymous and are detailed in Table 1. The three culture-adaptive substitutions present in the input S52 SGR sequence (AII) at the time of electroporation were maintained following selection, with a frequency of 100 % for all three (data not shown).

**Table 1.** Nucleotide substitutions identified during selection of the S52 SGR

Nucleotide substitution	Amino acid substitution (S52 individual protein numbering)	Amino acid substitution (S52 polyprotein numbering) (H77 polyprotein numbering)	% variant frequency	Location	Residue in JFH-1	Residue in Con1
G340C	VII3L	V1145L (1139)	24	NS3	V	V
A1143C	K380N	K1412N (1406)	25	NS3	A	K
C1940T	A15V	A1678V (1672)	33	NS4A	A	A
C1978T	H28Y	H1691Y (1685)	70	NS4A	R	R
A2114G	E19G	E1736G (1730)	34	NS4B	S	Q
T3048A	N69K	N2047K (2041)	41	NS5A	L	N
A3998G	Q386R	Q2364R (2350a)	24	NS5A	S	absent

**Fig. 2.**
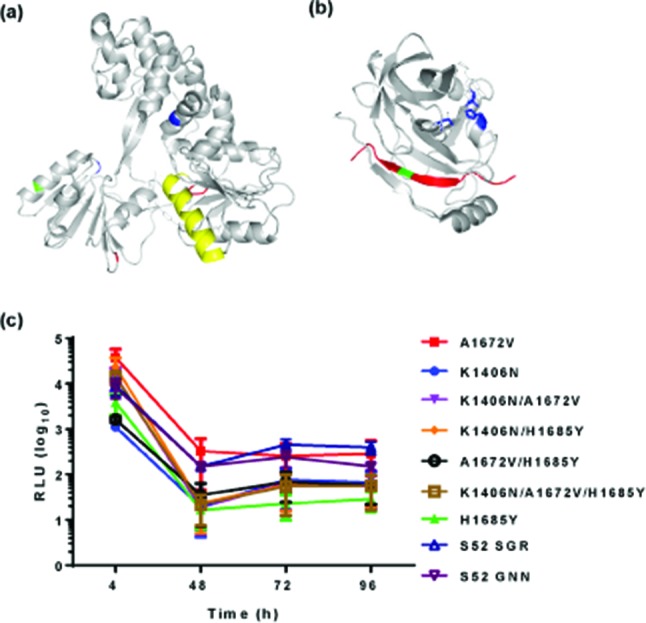
Culture-adaptive substitutions identified following selection of S52 SGR-harbouring cells. Location of putative culture-adaptive substitutions on three-dimensional structures of NS3 or NS4A. In (a) K1406 is shown in green within NS3 helicase. Proposed spring helix shown in yellow, nucleic acid binding cleft in blue and ATP-binding cleft shown in red. In (b) H1685 is shown in green within the NS3-binding region of the NS4A peptide (red), and the NS3 protease-active site shown in blue. A1672 is within the hydrophobic N terminus (not shown on crystal structure). (c) Transient replication of the S52 SGR culture-adaptive substitutions. The indicated substitutions were cloned back into S52 SGR, and 2 µg RNA transcripts were electroporated into Huh7.5 cells and harvested for luciferase assay. Luciferase activity is presented as absolute values. Error bars show the standard error of the mean of three experimental repeats.

Due to the lack of linkage of the short reads obtained by NGS, and the fact that the substitutions observed were not present in all reads, it was not possible to determine which combination(s) of substitutions might result in enhanced replication. To test this we performed an additional round of G418 selection in which we extracted total RNA from S52 SGR-harbouring cells and re-electroporated into naïve Huh7.5 cells. Despite detectable luciferase in these cells electroporated at 96 h post-electroporation (hpe) (data not shown), there was not sufficient material to amplify for further NGS. Therefore we subjected these cells to further selection with G418 as described previously. A large number of colonies were visible on these plates, which were pooled to give a polyclonal population. SGR RNA in these cells was again amplified by RT-PCR and subjected to NGS, and the only substitutions which were detected in this second round of sequencing were K1406N in NS3 and A1672V in NS4A (H77 numbering). Note that these substitutions are numbered K1412N and A1678V in S52 but will be referred to by H77 numbering henceforth, to comply with the accepted convention in the HCV field. We considered that these culture-adaptive substitutions might enable higher levels of transient replication of the S52 SGR and set out to test this hypothesis. We therefore introduced these substitutions back into the S52 SGR (AII) either singly or in combination. As H1685Y (H1691Y in S52) was the most prevalent substitution observed after the first round of selection (Table 1), we also included this substitution in our analysis. Fig. 2 shows the location of these putative culture-adaptive substitutions in the three-dimensional structures of the NS3 helicase and NS4A co-factor peptide bound to the active site of NS3 protease. The location of K1406N within the NS3 helicase domain and H1685Y within the NS4A co-factor peptide are highlighted in green; it was not possible to model A1672V as it is within the hydrophobic N-terminal domain. To act as a negative control we also generated a mutation in the active site of the NS5B RNA-dependent RNA polymerase (GNN) – this mutant has been shown in many SGRs to be replication inactive. As the transient replication of S52 SGR has been reported to be enhanced by the modification of the construct to include a firefly luciferase gene with reduced CpG and UpA dinucleotide frequencies [23], we also replaced the Feo reporter with a CpG/UpA-low firefly luciferase. All of these substitutions were compared to the parental S52 SGR and the GNN mutant. However, as shown in Fig. 2(c), none of these substitutions enabled the transient replication of the S52 SGR, although the single A1672V substitution in NS4A gave higher luciferase values than all the others.

### Robust transient replication of the S52 SGR also required modulation of the host cell environment

We considered that in addition to modifying the S52 SGR it might be possible to enhance transient replication by increasing the permissivity of the host cell for viral genome replication. To achieve this we evaluated two approaches: first, expression of the V protein from parainfluenza virus type 5 (PIV5) – a well-characterized interferon antagonist [24, 25]; and second, expression of the host cell protein SEC14L2 (also known as Tocopherol-associated protein, TAP1) [26]. SEC14L2 has been reported to enable replication of non-culture-adapted SGRs [26].

**Fig. 3.**
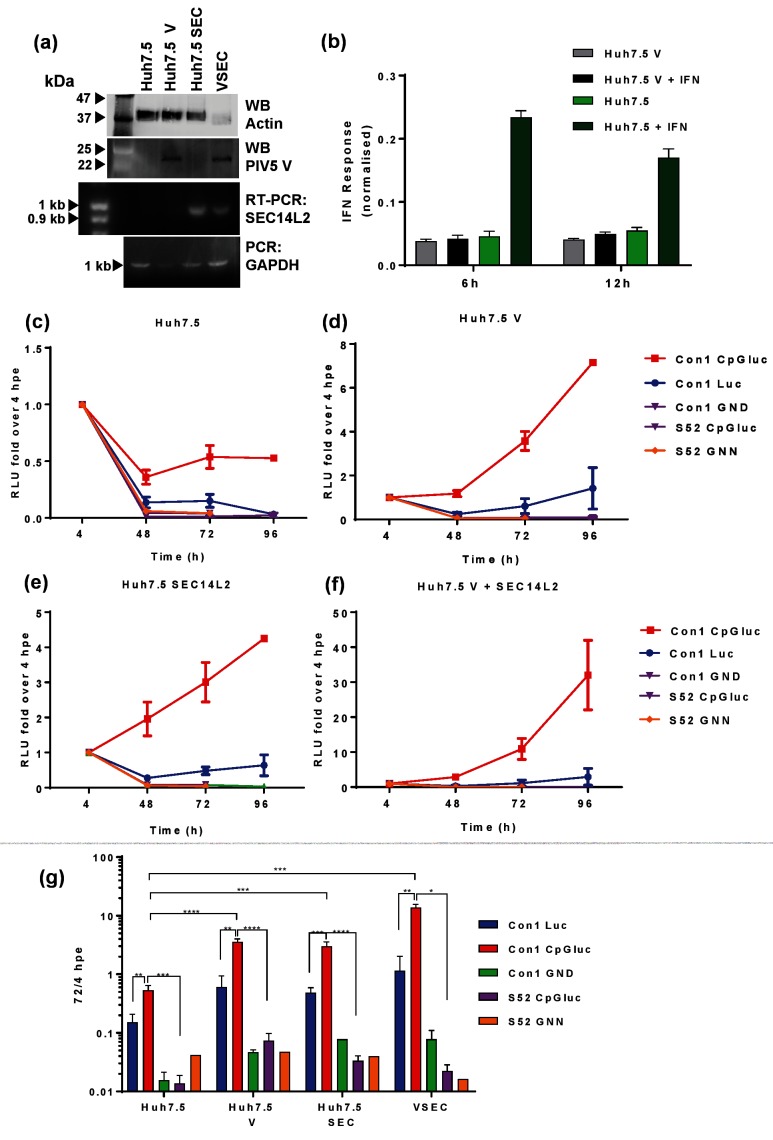
Enhancement of SGR replication using low-CpG luciferase, PIV-5 V protein and SEC14L2. (a) Confirmation of PIV-5 V or SEC14L2 expression by western blot or RT-PCR, respectively. (b) Huh7.5 or Huh7.5 V cells were transfected with an ISRE-luc plasmid and treated with IFN-α for 6 or 12 h prior to harvest for luciferase assay. Two micrograms of the indicated SGR RNA transcripts were electroporated into Huh7.5 (c), Huh7.5 V (d), Huh7.5 SEC14L2 (e) and Huh7.5 V+SEC14 L2 (termed VSEC cells) (f). (g) For comparative purposes all data were combined on to a single graph. Cells were harvested for luciferase assay at the indicated time points. Relative luciferase units are expressed as the ratio to 4 hpe. Error bars represent the standard error of the mean of four experimental repeats. **P≤0.01, ***P≤0.001, ****P≤0.0001.

We therefore established stable Huh7.5 cell lines expressing either the PIV5 V protein, SEC14L2 or both (the latter are termed VSEC cells hereafter). To verify the integrity of the cell lines, these were analysed by WB for PIV5 V protein expression and RT-PCR for SEC14L2 RNA (Fig. 3a). To confirm the expected activity of the PIV5 V protein, Huh7.5 and Huh7.5 V cells were transfected with an ISRE-luciferase construct and treated with IFN-α for 6 or 12 h. As expected there was an increase in luciferase in Huh7.5 cells treated with IFN-α, but this was not observed in Huh7.5 V cells (Fig. 3b), confirming that the PIV5 V protein abrogated IFN signalling.

**Fig. 4.**
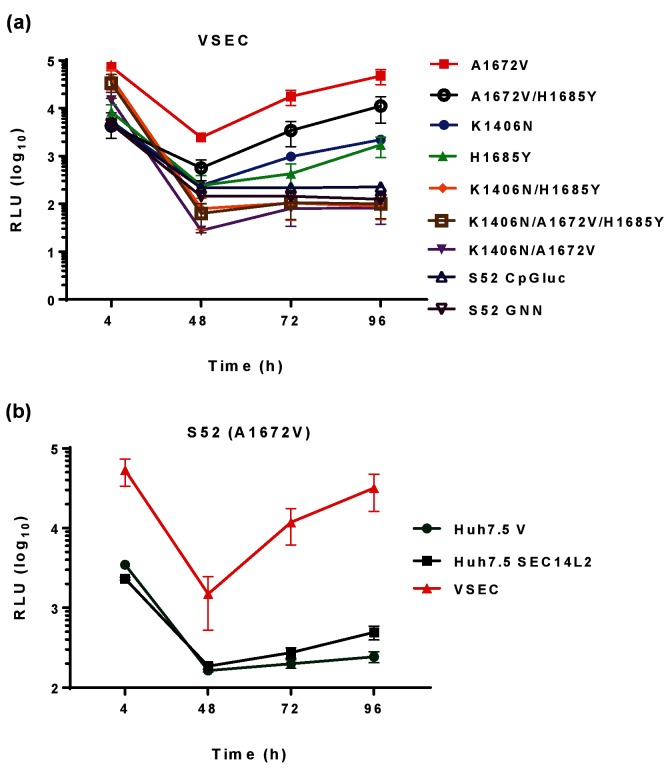
Replication of culture-adapted S52 SGR. (a) Two micrograms of the indicated RNA transcripts were electroporated into VSEC cells. (b) Two micrograms of S52(A1672V) RNA were electroporated into the indicated cell lines. Cells were harvested for luciferase assay at the indicated time points. Luciferase activity is presented as absolute values.

We then confirmed that these two proteins were able to enhance the transient replication of a GT1b (Con1) SGR. As expected [23], the CpG/UpA-low luciferase derivative of the Con1 SGR replicated better than a wild-type luciferase version in Huh7.5 cells (Fig. 3c). In addition, the presence of either PIV5 V or SEC14L2 enhanced replication of both the wild-type and CpG/UpA-low luciferase Con1 SGR (Fig. 3d, e), and the presence of both proteins had an additive effect (Fig. 3f). However, the presence of PIV5 V, SEC14L2 or both was not sufficient to support replication of the S52 SGR with CpG/UpA-low luciferase. We then tested whether any combination of the three culture-adaptive substitutions (K1406N, A1672V and H1685Y) were able to exhibit detectable transient replication of the S52 SGR in VSEC cells. As shown in Fig. 4, this was indeed the case. Compared to the observations in Huh7.5 cells (Fig. 2a) the NS4A substitution A1672V, either alone or in combination with the other NS4A substitution H1685Y, enhanced replication at 96 hpe by between 100–1000-fold. H1685Y alone, or the NS3 substitution K1406N alone, gave a 10-fold enhancement of replication. However, all other combinations did not replicate, exhibiting similar profiles to either the parental S52 SGR or the GNN NS5B mutant, suggesting that although these three substitutions were able to enhance replication there was some degree of incompatibility between them.

### Validation of the transient S52 SGR for DAA screening

**Fig. 5.**
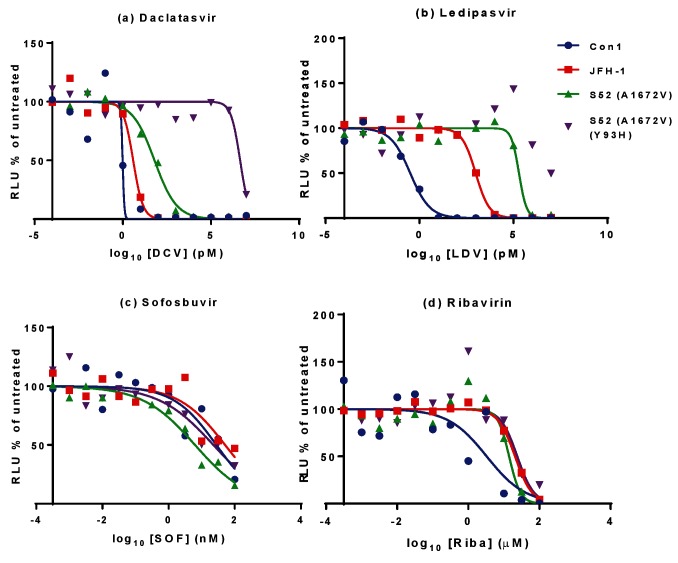
Effect of clinically approved anti-HCV compounds on S52 SGR replication. 8×10^3^ VSEC cells electroporated with either S52(A1672V), S52(A1672V)(Y93H), Con1 CpG/UpA-low SGR or SGR-Feo-JFH-1 were seeded into each well of a white 96-well plate. Cells were treated after either 4 h (Con1/JFH-1) or 24 h (S52), with the indicated concentrations of DCV (a), LDV (b), SOF (c) or ribavirin (d) for 72 h before being harvested for luciferase assay. DMSO vehicle concentration was 0.25 %. Relative luciferase units are expressed as a percentage of vehicle-only treated cells and 50 % effective concentrations calculated using Graphpad software.

The development of a transient S52 SGR opened up the possibility that this system could be used to screen for DAAs and/or investigate resistance. As proof of principle we therefore focused our attention on the most active of the three culture adaptive substitutions – A1672V. For clarity this SGR (which also contained the CpG/UpA-low luciferase) will be referred to as S52(A1672V). We also generated a further derivative of S52(A1672V) containing a Y93H (Y2065H by H77 polyprotein numbering) substitution within domain I of NS5A – this has previously been reported to result in DCV resistance in multiple GTs. S52(A1672V) and the Y93H derivative were electroporated into VSEC cells and then treated from 4 to 48 hpe with a range of concentrations of DCV, LDV, SOF or ribavirin (Fig. 5). As controls, VSEC cells were electroporated with either a CpG/UpA-low Con1 (GT1b) SGR [23] or SGR-Feo-JFH-1.

From the graphs, 50 % effective concentrations (EC_50_) were calculated using Graphpad Prism software and are presented in Table 2. As expected, S52(A1672V) was less sensitive to DCV or LDV, compared to both Con1 and JFH-1. In addition, the Y93H substitution resulted in an extraordinary 70 000-fold decrease in sensitivity to DCV; this difference was less pronounced for LDV as the wild-type S52(A1672V) SGR was already highly resistant to LDV. There was a modest difference in sensitivity of the SGR tested to SOF and ribavirin.

**Table 2.** EC_50_ values for transient SGR

		DCV	LDV	SOF	Ribavirin
Con1	EC_50_	0.98 pM	0.32 pM	27.9 nM	8.7 µM
JFH-1	EC_50_	3.9 pM	0.98 nM	51.2 nM	22.9 µM
	Fold over Con1	3.97	3038	1.83	6.27
S52	EC_50_	63 pM	200 nM	6.3 nM	12.6 µM
	Fold over Con1	64.2	614 000	0.23	4.27
S52 Y93H	EC_50_	4.5 µM	>40 µM	22.7 nM	23.9 µM
	Fold over Con1	4 600 000	nd	0.81	7.25
	Fold over S52	71 600	nd	3.6	1.7

nd, Not determined.

**Fig. 6.**
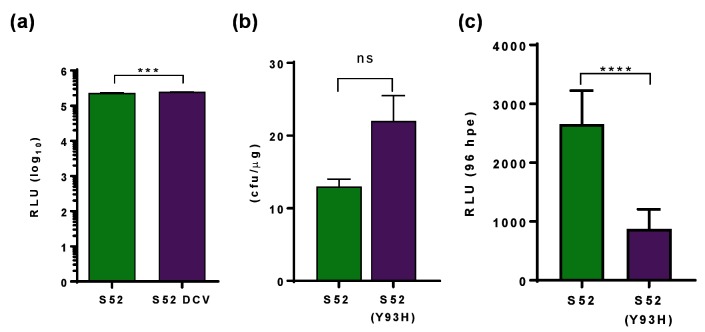
Comparison of transient and stable replication of S52 SGR. (a) Cells harbouring SGR-Feo-S52, or SGR-Feo-S52 selected with DCV, were seeded at a density of 8×10^3^ per well of a 96-well plate and harvested for luciferase assay after 72 h. Luciferase activity is presented as absolute values. (b) Two micrograms of RNA transcribed from SGR-neo-S52 or SGR-neo-S52(Y93H) were electroporated into Huh7.5 cells and seeded at a density of 2×10^5^ cells per well of a 6-well plate. Cells were selected with 0.5 mg ml^−1^ G418 from 48 hpe for three weeks before being fixed and stained with 0.5 % crystal violet in 10 % formalin and manually counted. (c) Two micrograms of RNA transcribed from S52(A1672V) or S52(A1672V) Y93H was electroporated into VSEC cells and 1×10^4^ cells were harvested for luciferase assay at 96 hpe. ***P≤0.001, ****P≤0.0001, ns: not significant.

Use of the transient system also revealed an additional aspect of DAA resistance: when the stable S52 SGR-harbouring cells were treated with DCV at a concentration equating to 100×EC_50,_ this resulted in the acquisition of a Y93H resistance-associated substitution (RAS) (as assessed by RT-PCR and Sanger sequencing – data not shown). The resulting DCV-resistant SGR replicated at a similar level to the wild-type SGR (Fig. 6a) and thus did not exhibit a fitness cost in acquiring DCV resistance. A similar observation was made when enumerating colony formation following electroporation of either SGR-Feo-S52 wild-type or Y93H (Fig. 6b). However, in the transient assay Y93H exhibited a significant fitness cost, replicating over 96 hpe at less than 50 % of wild type (Fig. 6c).

## Discussion

Transiently replicating SGRs have been instrumental in the elucidation of the functions of the HCV non-structural proteins, mechanisms of genome replication and development of DAAs. The three SGRs which have so far been reported for GT3, derived from two different isolates, are limited in their ability to replicate efficiently in a transient system and only replicate efficiently following selection with neomycin [16]. However, such stable replicon-harbouring cell systems are of limited use for development of DAAs and do not allow investigation into the mechanisms of resistance, since the most widely reported RAS within NS5A – Y93H – is associated with a fitness cost [27]. As they contain pre-existing active genome replication complexes they are not able to model early stages in the infectious cycle – namely translation of incoming genomic RNA, and subsequent establishment of replication complexes by the newly synthesized non-structural proteins. As such, they are a less representative model of HCV replication than transient SGRs. Transient replication of S310 was measured but showed luciferase levels of only several orders of magnitude lower than input translation at 4 hpe, and replication of the S52 SGR developed in transfected-and-cured Lunet cells did not replicate efficiently until 7 days post-transfection [17, 18].

The S52 SGR reported by Saeed *et al*. did not replicate transiently in our hands in Huh7.5 cells, which have a defect in innate intracellular immunity due to a mutation in RIG-I [28]. As reported [16], we were able to select stable SGR-harbouring cells using G418 and we identified an additional culture adaptation, A1672V in NS4A. When introduced into the S52 CpG/UpA-low luciferase SGR by site-directed mutagenesis, this conferred high levels of replication but only in cells expressing both PIV5 V and SEC14L2 (Fig. 4). The A1672V substitution is located in the hydrophobic N-terminal, membrane-anchoring domain. It is not clear why a substitution from a hydrophobic to polar amino acid side chain in this region proves to be so beneficial to replication. However, it is interesting to note that in a recombinant genome comprising the S52 5′UTR-NS5A with JFH-1-derived NS5B and 3′UTR (5-5A recombinant), A1672S is one of the three key mutations required for efficient replication, the others being F1464L in NS3 and D2979G in NS5B (the LSG combination) [29]. In contrast, the group of Jens Bukh also recently generated a full-length GT3a genome (DBN3a_cc_) which replicated as efficiently as JFH-1 [30], but this did not contain substitutions at any of the sites identified in our study; however, one of the 17 substitutions in this construct was Y1680C in NS4A. Clearly therefore, in GT3, NS4A is a hot-spot for culture-adaptive mutations pointing to a key role for this small protein in virus–host interactions.

We investigated a number of approaches to increase the replication fitness of the SGR or to modulate the host cell environment to increase permissibility to SGR replication. DNA from different types of organism differs in CpG and UpA dinucleotide frequency; in particular, as luciferase is insect-derived, it contains a higher frequency than mammalian genes. Optimization of CpG and UpA dinucleotide frequency increases the replication capacity of a number of viruses [31]. This effect is thought to be mediated by avoiding an as yet uncharacterized innate immune recognition of high-CpG/UpA sequences [32]. In our hands replacement of the Feo reporter cassette with a CpG/UpA-low luciferase did not by itself allow detectable replication of S52, despite conferring a fourfold increase in replication on Con1 SGR, although we note that others did observe an enhancement of S52 replication [23].

The parainfluenza virus type 5 (PIV5) V protein blocks STAT1-mediated immune activation by binding directly to STAT1 and inhibiting downstream interferon-α activation [25, 33, 34]. It has been shown that stable expression of the V protein enhances replication of HCV in human foetal liver cells [35]. Recently, the host cell protein SEC14L2 was found to allow replication of an unadapted SGR or isolates from patient samples including GT3. This is thought to work by accumulation of vitamin E, which provides protection against lipid peroxidation [26]. By combining both V and SEC14L2, together with a CpG/UpA-low luciferase and the A1672V culture adaptation in NS4A, we were able to establish a transiently replicating S52 SGR.

The utility of this transiently replicating GT3 SGR in antiviral development was tested by treatment with NS5A inhibitors DCV and LDV, NS5B inhibitor SOF and ribavirin, which is recommended alongside DAA combination therapies, particularly with respect to GT3 treatment. The NHS Extended Access Program found that the SVR achieved with a SOF/LDV combination compared to SOF/DCV was markedly less for GT3 patients [11]. SOF is reported to be pan-genotypic, and our data agree with this as we observed no differences in the EC_50_ for SOF between GT1, GT2 and GT3 SGRs (Fig. 5c). In contrast, and in agreement with clinical trial reports, we observed that the GT3 EC_50_ for DCV and LDV was significantly higher than for GT1, and the GT3 EC_50_ for LDV was several orders of magnitude higher than DCV. This also concurs with data published recently on the full-length GT3a genome (DBN3a_cc_), for which LDV was found to be significantly less effective [30]. DBN3a_cc_ was also more resistant to both DCV and LDV, compared to a GT1 virus. The results obtained here using a transient SGR system thus compare favourably to those obtained using full-length infectious virus assays. In addition, Y93H RAS in NS5A was significantly less sensitive to both NS5A inhibitors than wild-type S52. We demonstrated that Y93H was selected during passage of stable S52-harbouring cells in the presence of DCV. To the best of our knowledge this is the first such observation of selection for DCV resistance in a complete GT3 SGR *in vitro*, although Y93H was also selected following DCV treatment of a chimeric SGR containing a hybrid NS5A protein (amino acids 1–429 of GT3) in a GT2a (JFH-1) backbone [20]. In the latter study the authors also observed a modest fitness cost of the Y93H RAS and identified a second RAS, L31F, that was not seen in our study. We did not observe a Y93H-associated fitness cost in stable SGR-harbouring cells, but in the transient assay described here Y93H exhibited a lower replication capacity. Taken together, these observations suggest that development of resistance, and the corresponding fitness cost, depend on the sequence context, underscoring the importance of working with intact (i.e. non-chimeric) SGRs or infectious viruses.

This work details the development of an efficiently replicating GT3 SGR which can be applied to the discovery and development of combination therapies, analysis of resistance and further study into the differences between this and other genotypes, which may inform other aspects of HCV research.

## Methods

### Plasmids

S52 feo (AII variant) SGR was obtained from Charles Rice [16]. Con1 SGRs with wild-type or CpG/UpA-low luciferase were obtained from Peter Simmonds [23]. Insertion of CpG/UpA-low luciferase into S52 feo required the insertion of a unique AscI site, which resulted in mutation of the last residue of the 19-residue section of core protein, immediately upstream of the luciferase start codon, from proline to alanine; translation of the reporter was not compromised. Primer sequences available on request. Plasmid containing SEC14L2 for lentiviral transduction was obtained from Peter Simmonds. Site-directed mutagenesis was performed using the QuikChange protocol from Stratagene and sub-cloning was carried out according to standard techniques. Modified SGRs were verified by sequence analysis.

### Cell lines and reagents

Huh7.5 cells were maintained in DMEM containing 4.5 g l^−1^ glucose, 2 mM glutathione and sodium pyruvate (Lonza) supplemented with 10 % FBS (Sera Laboratories International), penicillin/streptomycin (Sigma) and non-essential amino acids (Lonza) in a humidified 37 °C, 5 % CO_2_ incubator. Huh7.5 cells stably transfected with parainfluenza virus 5 (PIV-5) V protein (Huh7.5-V) were obtained from Stephen Griffin (University of Leeds). Huh7.5-V cells were maintained in DMEM as described above with 0.5 mg ml^−1^ G418. Lentiviruses were generated using a plasmid containing SEC14L2 as reported [23], and transduced Huh7.5 and Huh7.5-V cell lines were were selected with 2 µg ml^−1^ puromycin (Huh7.5) or both G418 and puromycin (Huh7.5-V).

### RNA synthesis and SGR replication assay in cultured cells

SGR plasmids were linearized with *Xba*I (New England Biolabs) and RNA was transcribed using a T7 transcription kit (Promega) according to the manufacturer’s instructions. Two micrograms of RNA transcripts were electroporated into 2×10^6^ cells in diethyl pyrocarbonate (DEPC)-phosphate-buffered saline (PBS) using a square-wave protocol at 260 V for 25 ms. For selection of stable, SGR-harbouring cells, 10^6^ electroporated cells were seeded into 10 cm^2^ dishes and selected with 0.5 mg ml^−1^ G418 from 48 hpe. Surviving cells were pooled into polyclonal populations for further analysis. For replication assays, electroporated cells were seeded in white 96-well plates at a density of 10^4^ cells per well in four assay replicates for each. Following incubation, cells were washed with 1×PBS and lysed in 30 µl passive lysis buffer (PLB: Promega). Luciferase activity was measured on a FLUOROstar Optima plate reader (BMG Labtech) primed with Luciferase Assay Reagent I (LAR I, Promega).

### Immunoblotting

Cells were washed twice in PBS and lysed in Glasgow Lysis Buffer [GLB; 1 % (vol/vol) Triton X-100, 120 mM KCl, 30 mM NaCl, 5 mM MgCl_2_, 10 % (vol/vol) glycerol, 10 mM PIPES [piperazine-N,N′-bis(2-ethanesulfonic acid)]-NaOH, pH 7.2, with protease and phosphatase inhibitors], and clarified by centrifugation at 2800 ***g*** for 5 min at 4 °C. Protein concentration was measured for normalization by bicinchoninic acid assay (BCA, Pierce). Proteins were resolved on 7.5 % polyacrylamide gel before being transferred to polyvinylidene difluoride (PVDF) membrane. Membranes were blocked in 50 % (v/v) Odyssey blocking buffer (LI-COR) in Tris-buffered saline (TBS) and incubated in primary antibodies overnight at 4 ˚C and infra-red tagged secondary antibodies (LI-COR) at room temperature for 1 h. Primary antibodies used were anti-NS5A (sheep, 1 : 5000, [36]), anti-V5 tag (rabbit, 1 : 1000, Cell Signalling Technologies) and anti β-actin (mouse, 1 : 20 000, abcam). Secondary antibodies were anti-sheep, anti-rabbit (both 800 nm) and anti-mouse (680 nm), all used at 1 : 15 000. Membranes were imaged using a LI-COR Odyssey infra-red imaging system.

### Immunofluorescence

Cells were washed once in PBS, fixed for 10 min in 4 % (w/v) paraformaldehyde (PFA) and permeabilized in 0.1 % (v/v) Triton X-100 in PBS. Fixed cells were immunostained with anti-NS5A (as described above) at 1 : 2000 and anti-sheep (488 nm, AlexaFluor). Cells were mounted using Prolong Gold antifade mountant with DAPI and imaged using a Carl Zeiss LSM 700 inverted microscope and Zeiss Zen 2012 software.

### RNA extraction from cells and PCR

Stable SGR-harbouring cells were harvested in TRIZol (Invitrogen Life Technologies) and RNA purified according to the manufacturers’ instructions. One microgram of RNA was reverse-transcribed using Superscript II (Invitrogen) and random hexamer primers. Two microlitres of this cDNA were used as a template for PCR amplification of SEC14L2 or glyceraldehyde-3-phosphate dehydrogenase (GAPDH) as a loading control. DNA fragments were resolved on 1 % agarose gel.

### Next-generation sequencing (NGS)

Viral RNA was extracted from cells stably harbouring the S52 SGR using the RNeasy plus mini kit (Qiagen). PCR amplification and NGS was performed as previously described [37] with modifications. Briefly, the SGR was amplified using HCV GT3a genotype-specific primers for four overlapping amplicons spanning the HCV GT3a non-structural genes. The forward primers for the NS3_4A fragment were redesigned to be complementary to the EMCV IRES region of the SGR (primer sequences available upon request). Viral RNA was amplified by single-step RT-PCR (Superscript III Reverse Transcriptase, Invitrogen), followed by nested or semi-nested PCR. PCR products were purified using the QIAQuick kit (QIAGEN) and quantified by Qubit dsDNA Broad Range and High Sensitivity Assay Kits and the Qubit 2.0 Fluorometer (Life Technologies). Alternate amplicons were pooled in two reactions of equimolar amounts and 1 ng µl^−1^ of the pooled DNA was used for library preparation (Nextera XT DNA sample preparation kit; Illumina) according to manufacturer's instructions. Indexed libraries were sequenced using Illumina MiSeq deep sequencing reagent kit v2 (Illumina).

### Treatment with antiviral compounds

VSEC cells (described above) were electroporated with 2 µg SGR RNA transcripts, and 10^4^ electroporated cells were seeded into white 96-well plates. Cells electroporated with Con1 and JFH-1 were treated with the indicated concentrations of DCV, LDV (both SelleckChem), SOF (Gilead) or ribavirin (Sigma) in duplicate with 0.25 % (v/v) final DMSO at 4 hpe for 72 h. Cells electroporated with S52 wild-type and Y93H were treated at 24 hpe for 72 h, following a preliminary observation that replication is not reliably detected until 96 hpe. This allowed a constant treatment duration of 72 h for all electroporated cells. Cells were harvested for luciferase after 72 h treatment as described above.
